# Development of Melanocortin 4 Receptor Agonists by Exploiting Animal-Derived Macrocyclic, Disulfide-Rich Peptide Scaffolds

**DOI:** 10.1021/acsptsci.3c00090

**Published:** 2023-09-26

**Authors:** Edin Muratspahić, Despoina Aslanoglou, Andrew M. White, Claudia Draxler, Xaver Kozisek, Zara Farooq, David J. Craik, Peter J. McCormick, Thomas Durek, Christian W. Gruber

**Affiliations:** † Center for Physiology and Pharmacology, Institute of Pharmacology, Medical University of Vienna, 1090 Vienna, Austria; ‡ Institute for Molecular Bioscience, Australian Research Council Centre of Excellence for Innovations in Peptide and Protein Science, The University of Queensland, Brisbane, Queensland 4072, Australia; § Department of Endocrinology, Queen Mary University of London, London E1 4NS, U.K.

**Keywords:** melanocortin 4 receptor, nature-derived peptide, disulfide-rich, molecular grafting

## Abstract

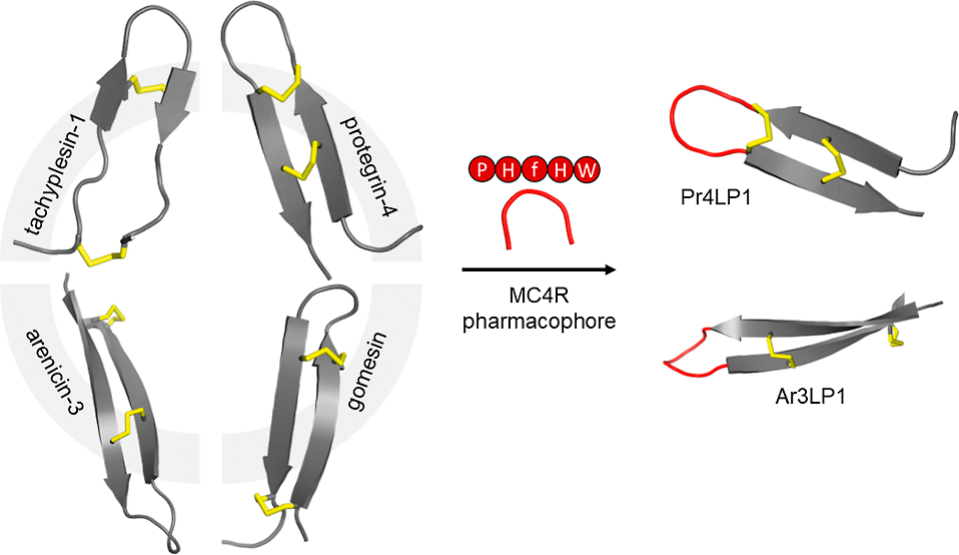

G protein-coupled receptors are among the most widely studied classes of drug targets. A major challenge in this field is to develop ligands that will selectively modulate a single receptor subtype to overcome the disadvantages of undesired “off target” effects caused by lack of target and thus signaling specificity. In the current study, we explored ligand design for the melanocortin 4 receptor (MC4R) since it is an attractive target for developing antiobesity drugs. Endogenously, the receptor is activated by peptide ligands, i.e., three melanocyte-stimulating hormones (α-MSH, β-MSH, and γ-MSH) and by adrenocorticotropic hormone. Therefore, we utilized a peptide drug design approach, utilizing “molecular grafting” of pharmacophore peptide sequence motifs onto a stable nature-derived peptide scaffold. Specifically, protegrin-4-like-peptide-1 (Pr4LP1) and arenicin-1-like-peptide-1 (Ar3LP1) fully activated MC4R in a functional cAMP assay with potencies of 3.7 and 1.0 nM, respectively. In a nanoluciferase complementation assay with less signal amplification, the designed peptides fully recruited mini-Gs with subnanomolar and nanomolar potencies. Interestingly, these novel peptide MC4R ligands recruited β-arrestin-2 with ∼2-fold greater efficacies and ∼20-fold increased potencies as compared to the endogenous α-MSH. The peptides were inactive at related MC1R and MC3R in a cAMP accumulation assay. These findings highlight the applicability of animal-derived disulfide-rich scaffolds to design pathway and subtype selective MC4R pharmacological probes. In the future, this approach could be exploited to develop functionally selective ligands that could offer safer and more effective obesity drugs.

The melanocortin receptors (MC1R-MC5R) are a prototypic class A family of G protein-coupled receptors (GPCRs). They govern a range of fundamental physiological processes and the ways in which they promote their cellular signaling events have been the focus of numerous biomedical research studies over many years.^1^ Melanocortin 4 receptor (MC4R) is among the most extensively studied GPCR, and its key regulation of energy homeostasis and appetite mediated by virtue of its signaling in the paraventricular nucleus of the hypothalamus makes it a promising therapeutic target for energy balance disorders, including obesity and cancer cachexia.^2,3^ In humans, loss-of-function mutations in MC4R have been linked to early onset severe obesity—the most common form of monogenic obesity^4^—while gain-of-function mutations have been associated with a low body-mass-index.^5,6^ In addition to its hypothalamic regulation of food intake and energy expenditure, MC4R has been implicated in blood pressure control,^7^ nociception,^8^ sexual function,^9^ and anhedonia.^10^ These diverse functional properties have expanded the therapeutic potential of MC4R for the development of drugs for the treatment of anxiety, depression, and sexual dysfunction.

MC4R and its closely related MCRs are activated by endogenous melanocortin peptides, three melanocyte-stimulating hormones (α-MSH, β-MSH, and γ-MSH), and adrenocorticotropic hormone.^11^ MC4R and MC3R are unique among GPCRs in that they can also be antagonized by the endogenous agouti-related peptide (AgRP).^11^ Extensive structure–activity relationship studies have identified a minimal tetrapeptide sequence motif (His–Phe–Arg–Trp, HFRW) of the melanocortin peptides that is required for their pharmacological activity at MCRs.^12^ Accordingly, the conserved “HFRW” pharmacophore has been extensively utilized to develop ligands targeting MCRs for therapeutic benefit,^13^ In particular, the macrocyclization of peptides possessing the HFRW pharmacophore has proven to be an effective strategy for developing potent peptide-based ligands of MCRs.^13^ Recent clinical approvals of the macrocyclized bremelanotide and setmelanotide for the treatment of hypoactive sexual desire disorder^14^ and severe obesity due to pro-opiomelanocortin or leptin receptor deficiency,^15^ respectively, have further energized efforts for the engineering of potent macrocyclized peptide ligands targeting MCRs.

The grafting of bioactive epitopes onto plant-derived cyclic, disulfide-rich peptide frameworks has been an effective strategy to obtain potent, selective, and stable MCR ligands. For instance, incorporating the HFRW motif into the stable 29-residue cyclotide scaffold resulted in a selective MC4R ligand with low nanomolar affinity and submicromolar potency.^16^ Harnessing the smaller disulfide-bridged 14-residue cyclic peptide from sunflower, i.e., sunflower trypsin inhibitor-1 (SFTI-1) as a template facilitated the development of peptide agonists with picomolar potency and selectivity toward MC1R.^17,18^ White et al. later exploited SFTI-1-based late-stage functionalization with cysteine staples to generate selective MC1R agonists with desired pharmacological properties.^19^

Inspired by these molecular grafting studies, we sought to expand the existing repertoire of macrocyclic selective MC4R agonists. Our rationale was to design these novel peptide probes by grafting the HFRW pharmacophore into other disulfide-rich (in this case animal-derived) peptide frameworks and test their pharmacological properties at MC4R. We synthesized 14 macrocyclic peptide analogues based on the antimicrobial peptides tachyplesin-1, protegrin-4, arenicin-3, and gomesin and elucidated their functional activity at MC4R. Our findings demonstrate that animal-derived macrocyclic, disulfide-rich antimicrobial peptides are valuable molecular frameworks to design potent and selective MC4R agonists. Such stabilized peptide scaffolds can be exploited to design potent peptide-based ligands to unravel the MC4R pharmacology. These novel GPCR-targeting peptides could serve as excellent starting points to develop antiobesity medications targeting MC4R.

## Results

### Design and Synthesis of MC4R Ligands Based on Animal-Derived Disulfide-Rich Scaffolds

The majority of disulfide-rich scaffolds utilizing molecular grafting for the development of MC4R ligands until now were derived from plants.^20^ To identify novel disulfide-bridged frameworks, here we focused on the structural diversity of antimicrobial peptides of animal origin.^21^ Specifically, we leveraged tachyplesin-1, protegrin-4, arenicin-3, and gomesin templates on the basis of their β-hairpin structure connected by a β-turn that is further stabilized by two disulfide bonds.^21,22^ Furthermore, these scaffolds comprise a cluster of hydrophobic and positively charged amino acid residues that could engage in additional receptor interactions, thereby contributing to increased potency and affinity. Indeed, recently reported cryo-electron microscopy structures of MC4R bound to setmelanotide revealed that a basic arginine residue outside the HFRW core motif forms extensive electrostatic and hydrophobic interactions with receptor residues in the transmembrane domain.^23,24^ These receptor-specific interactions contribute to the high potency of setmelanotide.

**Figure 1:**
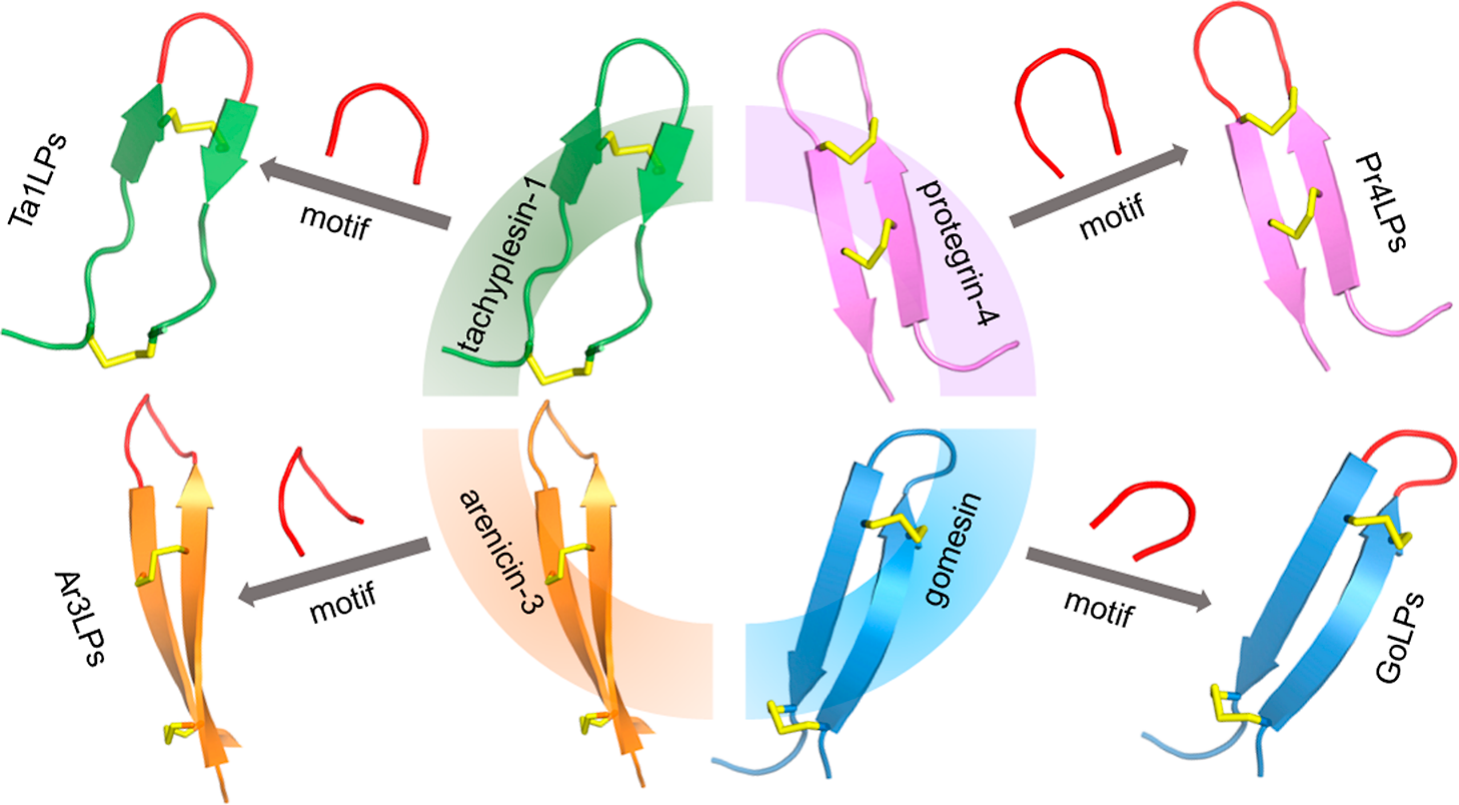
Molecular grafting approach. PHfHW, HfHW, and HfRW motifs (colored in red) were grafted onto scaffolds by replacing their native sequences (colored in green, pink, orange, and blue, respectively), resulting in the generation of tachyplesin-1-like peptides (Ta1LPs), protegrin-4-like peptides (Pr4LPs), arenicin-3-like peptides (Ar3LPs), and gomesin-like peptides (GoLPs). Disulfide bonds are highlighted in yellow. Tachyplesin-1 (PDB: 1MA2), protegrin-4 (PDB: 6QKF), arenicin-3 (PDB: 5V0Y), and gomesin (1KFP). The structural cartoons are shown for illustration only and are not accurate representations of the grafted peptides.

**Figure 2:**
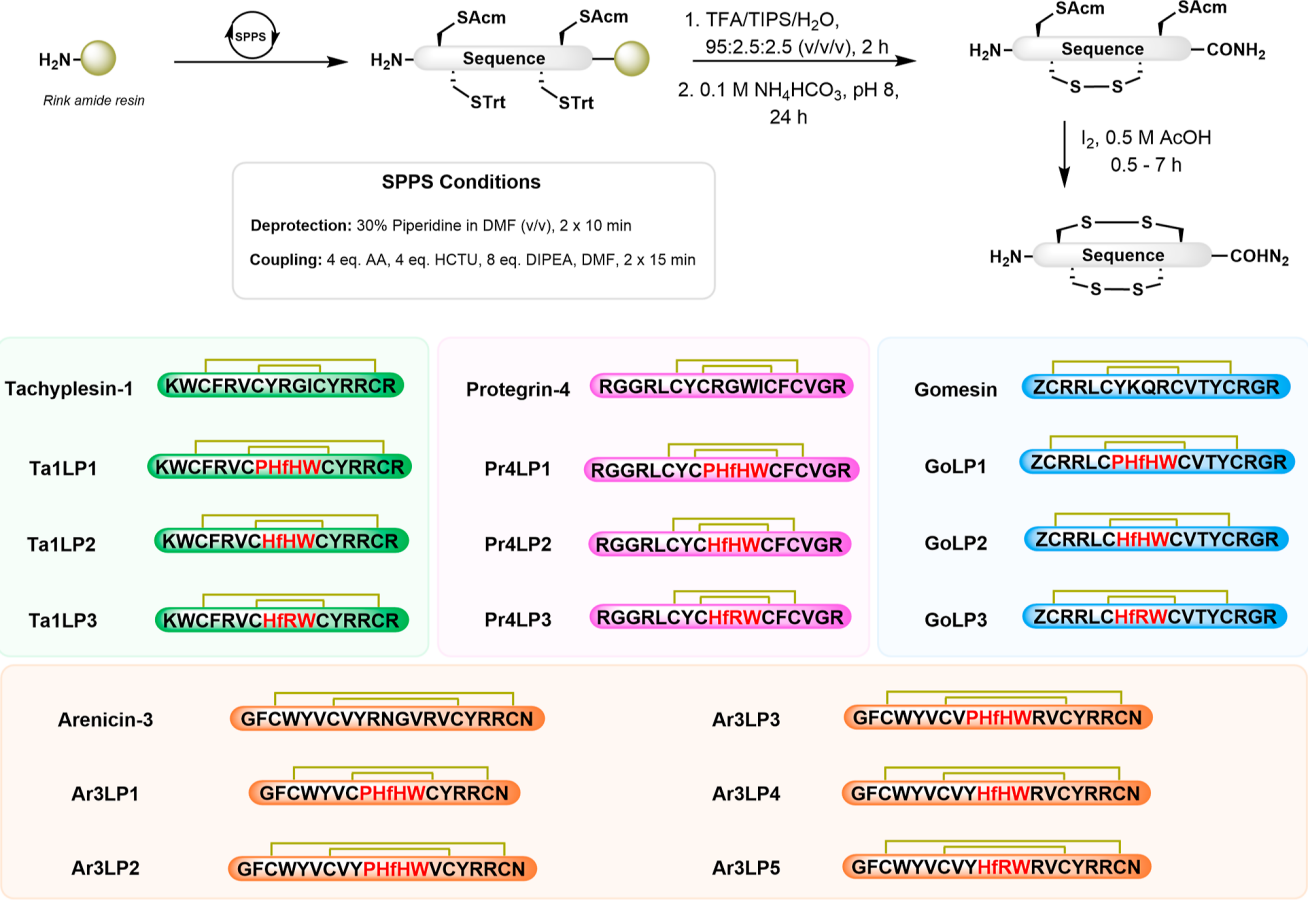
Overview of the synthetic strategy to create grafted peptides. Ta1LP: tachyplesin-1-like peptide (green); Pr4LP: protegrin-4-like peptide (pink); Ar3LP: arenicin-3-like peptide (orange); GoLP: gomesin-like peptide (blue); Acm: acetamidomethyl; Trt: trityl. For details and synthesis conditions, please refer to the Materials section.

Based on this recent structural knowledge, we replaced the turn region of the native sequences of our chosen scaffolds by each of the three pharmacophores, Pro–His–d-Phe–His–Trp (PHfHW), His–d-Phe–His–Trp (HfHW), or His–d-Phe–Arg–Trp (HfRW) and flanked them by an interstrand disulfide bridge: Cys7–Cys12 for tachyplesin-1, Cys8–Cys13 for protegrin-4, Cys7–Cys16 for arenicin-3, and Cys6–Cys11 for gomesin (Figure 1, Table 1). These pharmacophores have potent activity at MC4R.^17,25^ We chose to use d-Phe instead of l-Phe in the pharmacophore sequences because its insertion in previous studies led to an increase in potency.^26^ In addition, since the native sequence of arenicin-3 between Cys7 and Cys16 contains eight amino acid residues, we designed five analogues either by replacing the entire native sequence by the pentapeptide pharmacophore or tetra- and penta-peptide motifs were flanked by the remaining native amino acids (Table 1). Following the design process, the peptides Ta1LP (tachyplesin-1-like peptide), Pr4LP (protegrin-4-like peptide), Ar3LP (arenicin-3-like peptide), and GoLP (gomesin-like peptide) were produced by Fmoc-based solid-phase peptide synthesis using strategically placed acetamidomethyl (Acm) and trityl (Trt) protecting groups for the four cysteines (Cys) to precisely direct formation of the two disulfide bonds (Figures 2, S1–S4 and Table S1).

**Table 1:** Sequences and Pharmacological Properties of Designed MC4R Peptide Ligands

scaffold	ligand	sequence	potency cAMP		efficacy cAMP
			EC_50_ ± SD (M)	logEC_50_ ± SD (M)	*E*_max_ ± SD (%)
α-MSH	NDP-α-MSH	BYSXEHfRWGKPV-NH_2_^a^	0.4 ± 0.2 × 10^–^^10^	–10.4 ± 0.1	100
tachyplesin-1	native	KWC_3_FRVC_7_YRGIC_12_YRRC_16_R-NH_2_	n.d.	n.d.	n.d.
	Ta1LP1	KWC_3_FRVC_7_**PHfHW**C_13_YRRC_17_R-NH_2_	2.0 ± 1.4 × 10^–^^8^	–7.7 ± 0.1	97 ± 4
	Ta1LP2	KWC_3_FRVC_7_**HfHW**C_12_YRRC_16_R-NH_2_	4.8 ± 4.0 × 10^–^^8^	–7.3 ± 0.2	97 ± 6
	Ta1LP3	KWC_3_FRVC_7_**HfRW**C_12_YRRC_16_R-NH_2_	1.7 ± 1.1 × 10^–^^8^	–7.8 ± 0.1	98 ± 4
protegrin-4	native	RGGRLC_6_YC_8_RGWIC_13_FC_15_VGR-NH_2_	n.d.	n.d.	n.d.
	Pr4LP1	RGGRLC_6_YC_8_**PHfHW**C_14_FC_16_VGR-NH_2_	3.7 ± 0.7 × 10^–^^9^	–8.4 ± 0.1	96 ± 3
	Pr4LP2	RGGRLC_6_YC_8_**HfHW**C_13_FC_15_VGR-NH_2_	1.5 ± 1.0 × 10^–^^7^	–6.8 ± 0.1	97 ± 4
	Pr4LP3	RGGRLC_6_YC_8_**HfRW**C_13_FC_15_VGR-NH_2_	2.1 ± 1.5 × 10^–^^7^	–6.7 ± 0.1	99 ± 6
arenicin-3	native	GFC_3_WYVC_7_VYRNGVRVC_16_YRRC_20_N-NH_2_	n.d.	n.d.	n.d.
	Ar3LP1	GFC_3_WYVC_7_**PHfHW**C_13_YRRC_17_N-NH_2_	1.0 ± 0.1 × 10^–^^9^	–9.0 ± 0.1	98 ± 4
	Ar3LP2	GFC_3_WYVC_7_VY**PHfHW**VC_16_YRRC_20_N-NH_2_	1.8 ± 0.5 × 10^–^^8^	–7.7 ± 0.2	95 ± 10
	Ar3LP3	GFC_3_WYVC_7_V**PHfHW**RVC_16_YRRC_20_N-NH_2_	1.0 ± 0.3 × 10^–^^6^	–6.0 ± 0.3	117 ± 18
	Ar3LP4	GFC_3_WYVC_7_VY**HfHW**RVC_16_YRRC_20_N-NH_2_	8.2 ± 0.1 × 10^–^^7^	–6.1 ± 0.2	106 ± 14
	Ar3LP5	GFC_3_WYVC_7_VY**HfRW**RVC_16_YRRC_20_N-NH_2_	2.6 ± 2.1 × 10^–^^7^	–6.6 ± 0.2	99 ± 8
gomesin	native	ZC_2_RRLC_6_YKQRC_11_VTYC_15_RGR-NH_2_	n.d.	n.d.	n.d.
	GoLP1	ZC_2_RRLC_6_**PHfHW**C_12_VTYC_16_RGR-NH_2_	2.6 ± 1.6 × 10^–^^8^	–7.6 ± 0.1	99 ± 3
	GoLP2	ZC_2_RRLC_6_**HfHW**C_11_VTYC_15_RGR-NH_2_	1.5 ± 0.7 × 10^–^^7^	–6.8 ± 0.2	116 ± 11
	GoLP3	ZC_2_RRLC_6_**HfRW**C_11_VTYC_15_RGR-NH_2_	3.9 ± 2.4 × 10^–^^8^	–7.4 ± 0.1	104 ± 5

a For NDP-α-MSH, ‘B’ denotes acetylated N-terminal serine and X norleucine. Disulfide bonds 0 are formed between Cys3 and Cys16/17, Cys7 and Cys12/13 for tachyplesin-1; Cys6 and Cys15/16, Cys8 and Cys13/14 for protegrin-4; Cys3 and Cys20/17, Cys7 and Cys16/13 for arenicin-3; and Cys2 and Cys15/16, Cys6 and Cys11/12 for gomesin. f indicates d-Phe while Z denotes pyroglutamic acid. Incorporated pharmacophores are in bold and underlined. Pharmacological cAMP data are mean ± SD from two to three independent experiments (technical triplicates each). n.d., not determined.

### Pharmacological Evaluation of Ta1LP, Pr4LP, Ar3LP, and GoLP Ligands at MC4R in cAMP and BRET β-Arrestin-2 Recruitment Assays

**Figure 3:**
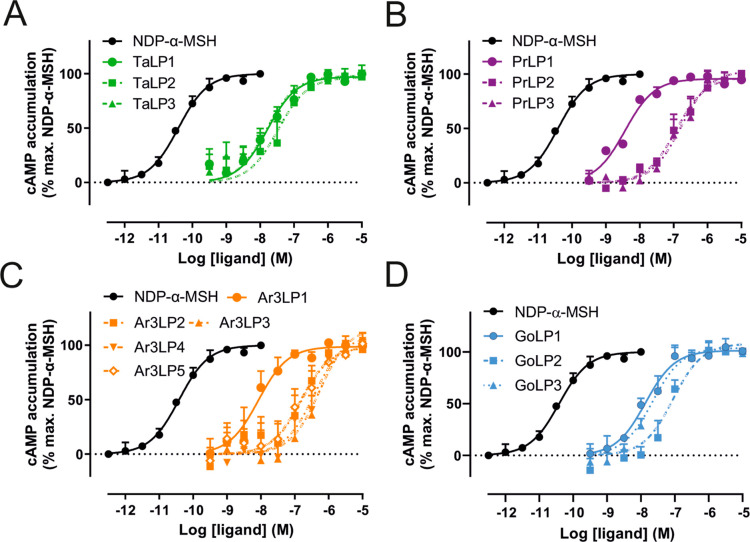
Functional cAMP assay of synthesized peptide analogues targeting MC4R. The accumulation of adenylyl cyclase-catalyzed cAMP of (A) tachylplesin-1-like peptides (Ta1LPs, green), (B) protegrin-4-like peptides (Pr3LPs, pink), (C) arenicin-3-like peptides (Ar3LPs, orange), and (D) gomesin-like peptides (GoLPs, blue) was measured in HEK293 cells transiently expressing mouse melanocortin 4 receptor (MC4R). NDP-α-MSH was used as a positive control. Data are from three independent experiments and are shown as mean ± SEM.

We explored the ability of the synthesized disulfide-rich peptides to activate MC4R by measuring adenylyl cyclase-dependent accumulation of cAMP. Eight of the 14 peptides displayed low nanomolar potencies (ranging from 1 to 48 nM); six peptides had potencies in the high nanomolar/low micromolar range (Table 1). All peptides were full agonists at MC4R, with *E*_max_ values comparable to the positive control NDP-α-MSH. For the tachyplesin-1 and protegrin-4 scaffolds, Ta1LP3 and Pr4LP1 exhibited similar potencies, with EC_50_ values of 16.7 and 3.7 nM, respectively (Figure 3A,B, Table 1). For the arenicin-3- and gomesin-based peptides, Ar3LP1 and GoLP1 elicited the most potent agonism at MC4R, with EC_50_ values of 1.0 and 26.0 nM, respectively (Figure 3C,D, Table 1). The animal-derived scaffold peptides without the pharmacophore inserts have not been assayed for activity at MC4R here, justified by the observation that in previous grafting studies, other scaffolds, e.g., the plant-derived SFTI-1 scaffolds were found to be inactive at melanocortin receptors.^17,18^

**Figure 4:**
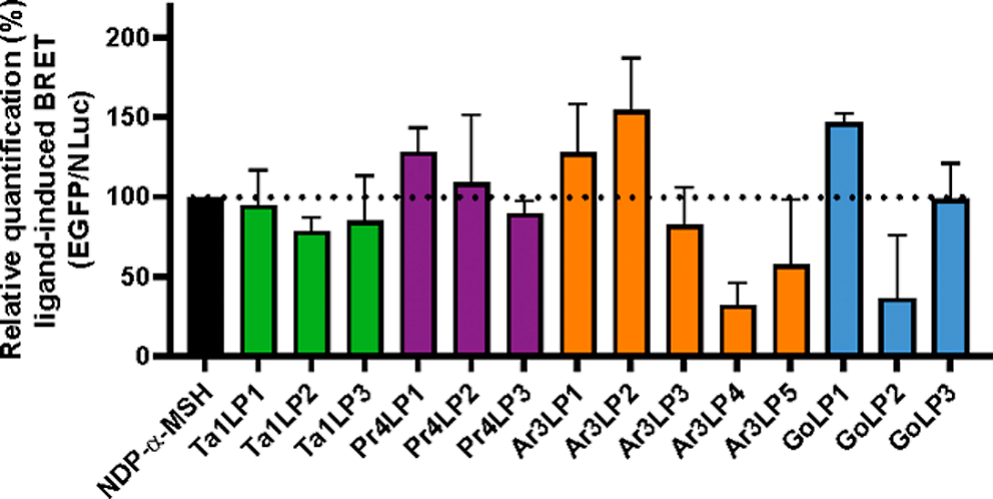
β-Arrestin-2 recruitment of designed MC4R peptide analogues. BRET assay was used to measure the transient interaction of MC4R-EGFP with β-arrestin-2-nanoluciferase (NLuc) in HEK293 cells over time following cell treatment with 10 μM of tachyplesin-1-like peptides (Ta1LPs, green), protegrin-4-like peptides (Pr4LPs, pink), arenicin-3-like peptides (Ar3LPs, orange), and gomesin-like peptides (GoLPs, blue). Relative BRET quantification was determined by calculating the mean value of ligand-induced BRET for each peptide ligand from 312 to 2433 s which was then divided by the mean value of ligand-induced BRET of NDP-α-MSH set as 100. Data are shown as mean ± SEM from three independent experiments.

Considering the physiological relevance of β-arrestins and their critical role in orchestrating MC4R signaling, we measured β-arrestin-2 recruitment in a kinetic bioluminescence resonance energy transfer (BRET) assay. We screened at a ligand concentration of 10 μM in HEK293 cells transiently expressing MC4R coupled to EGFP, and β-arrestin-2 fused to nanoluciferase. Ta1LPs recruited β-arrestin-2 similar to NDP-α-MSH (Figures 4, S5A), whereas Pr4LPs, Ar3LPs, and GoLPs displayed varying levels of BRET efficiency in recruiting β-arrestin-2 (Figures 4, S5B–D). For instance, Ar3LP4, Ar3LP5, and GoLP2 exhibited diminished β-arrestin-2 recruitment with BRET efficacies less than 60% compared to the positive control NDP-α-MSH (Figures 4, S5C,D). By contrast, Pr4LP1, Ar3LP1, Ar3LP2, and GoLP1 recruited β-arrestin-2 with greater efficiencies (>130%) than NDP-α-MSH (Figures 4 and S5B,C).

### Pharmacological Analysis and Validation of Pr4LP1 and Ar3LP1 Peptides as Potent Agonists at MC4R Using nanoluciferase Complementation G_s_ and β-Arrestin-2 Recruitment Assays

**Figure 5:**
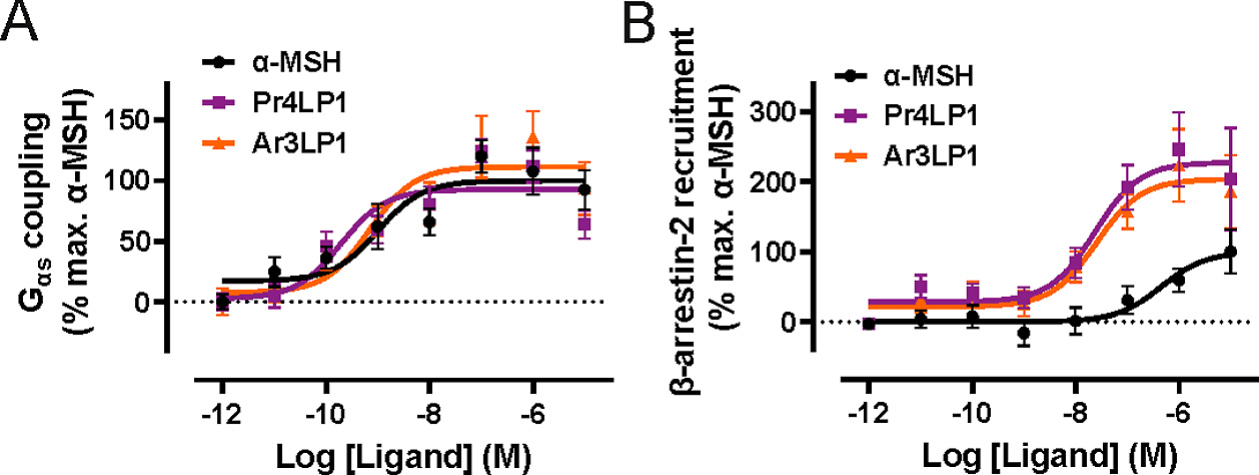
Biosensor characterization of Pr4LP1 and Ar3LP1 peptides acting as agonists at MC4R. (A) Nanoluciferase complementation assay between MC4R-LgBit and G_αs_-SmBit confirmed that both Pr4LP1 (EC_50_ = 193 pM) and Ar3LP1 (EC_50_ = 702 pM) peptides can mediate G_αs_ protein coupling to the MC4R, more potently than α-MSH (EC_50_ = 1 nM), and more efficaciously, with Pr4LP1 showing an 8.8% increase and Ar3PL1 showing a 25% increase in efficacy compared to α-MSH. (B) Nanoluciferase complementation between MC4R-SmBit and β-arrestin2-LgBit demonstrated that both Pr4LP1 and Ar3LP1 are markedly more efficacious than α-MSH, as Pr4LP1 showed 99% increase and Ar3LP1 showed 82% increase in efficacy (*E*_max_) compared to α-MSH, in mediating β-arrestin2 recruitment to the receptor. The two peptides also demonstrated increased potency (Pr4LP1: EC_50_ = 23.5 nM; Ar3LP1: EC_50_ = 25.2 nM) compared to that of the endogenous ligand (EC_50_ = 450 nM). Data are represented as means ± SEM for all experimental replicates and were performed in triplicate from *n* ≥ 3 independent experiments.

Our initial screening steps guided the selection of two MC4R-stimulating peptides, Pr4LP1 and Ar3LP1, with the highest efficacy in β-arrestin-2 recruitment and acceptable synthesis yields as compared to the other peptides. We proceeded with more detailed pharmacological characterization of Pr4LP1 and Ar3LP1 to gain more insights into the signaling pathways that follow MC4R stimulation. Using a live-cell based nanoluciferase complementation assay, between MC4R-LgBit and G_αs_-SmBit, we confirmed the ability of the peptides to directly stimulate MC4R and allow G_αs_ coupling to the receptor, which is in agreement with our initial cAMP second messenger quantification assay. Both peptides had slightly increased potencies in G_αs_ coupling (Pr4LP1 EC_50_ = 193 pM; Ar3LP1 EC_50_ = 702 pM) as compared to endogenous ligand α-MSH (EC_50_ = 1 nM) (Figure 5A). In addition to the improved potencies, both peptides displayed slightly increased efficacy in recruiting G_αs_ compared to α-MSH (Pr4LP1 8.8% and Ar3LP1 25% more efficacious than α-MSH), suggesting that they are full agonists at MC4R.

We utilized a similar luciferase complementation assay between MC4R-SmBit and β-arrestin-2-LgBit to study recruitment of β-arrestin-2 to the MC4R, in response to Pr4LP1 and Ar3LP1 and compared to α-MSH-mediated modulation. Interestingly, both peptides potently allowed β-arrestin-2 recruitment to MC4R (Pr4LP1 EC_50_ = 23.5 nM; Ar3LP1 EC_50_ = 25.2 nM) (Figure 5B); this corresponds to approximately 20-fold increased potency as compared to α-MSH (EC_50_ = 450 nM). Furthermore, both peptides demonstrated a marked increase in efficacy with Pr4LP1 showing 99% increase and Ar3LP1 demonstrating 82% increase in efficacy compared to the endogenous ligand α-MSH (Figure 5B). In an attempt to quantify signaling bias using the Black–Leff operational model by calculating ΔΔlog(τ/*K*_A_),^27–29^ we identified a trend for preferred β-arrestin-2 recruitment over Gα_s_ coupling, albeit not significant (data not shown).

### Selectivity Determination of Pr4LP1 and Ar3LP1 Peptides at MC1-, MC3-, and MC4R Using a pGlo cAMP Accumulation Assay

**Figure 6:**
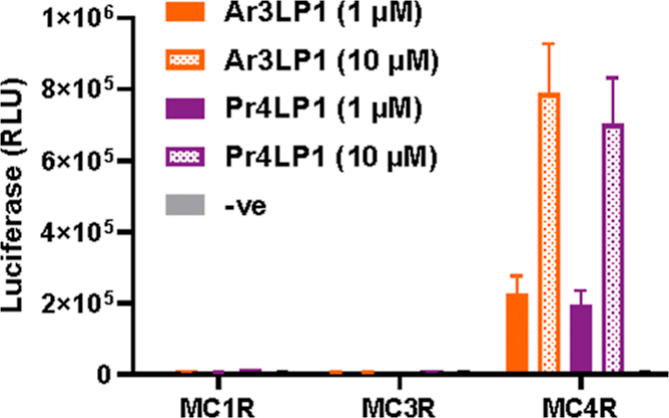
Selectivity determination of Pr4LP1 and Ar3LP1 peptides at MC4R vs MC1R and MC3R. A pGlo cAMP assay was used to demonstrate the effects of peptides Pr4LP1 and Ar3LP1 at the human MC1R, MC3R, and MC4R. Measurements exhibited no response (random luciferase units, RLU) at MC1R and MC3R for both peptides, whereas Pr4LP1 and Ar3LP1 (1 and 10 μM) activate the receptor. Data (not normalized) are represented as mean ± SEM from three independent experiments performed in technical triplicate.

Following functional analysis at MC4R, we were intrigued to know how selective Pr4LP1 and Ar3LP1 were for MC4R vs other melanocortin receptors. Using a pGlo cAMP accumulation assay, we determined that both peptides were only effective in activating MC4R and do not show a response at MC1R and MC3R up to concentrations of 10 μM (Figure 6). Albeit to be confirmed with binding studies in the future, this result suggests selective action at MC4R vs MC1R and MC3R.

## Discussion

In this study, we used animal-derived macrocyclic disulfide-rich antimicrobial peptide scaffolds to design agonists for MC4R with increased selectivity over other melanocortin receptors. The most potent analogues, Pr4LP1 and Ar3LP1, displayed high agonist potency at MC4R and a marked increase in efficacy toward β-arrestin-2 recruitment over the Gα_s_ protein signaling pathway, thus highlighting the potential of animal-derived macrocyclic, disulfide-rich frameworks for developing functionally selective ligands at MC4R in the future. Significantly, the two peptide analogues, Pr4LP1 and Ar3LP1, were selective for MC4R over MC1R and MC3R, in recruiting β-arrestin-2 (Figure S6).

Previously, engineering of MCR-targeting peptide ligands focused on plant-derived cyclic scaffolds and led to the generation of potent and selective melanocortin receptor agonists. Exploiting the cyclotide scaffold, for instance, enabled the design of a selective MC4R ligand with low nanomolar affinity,^16^ whereas using the SFTI-1 template facilitated the engineering of peptide agonists with picomolar potency and selectivity toward MC1R.^17,18^ Herein, we broadened the applicability of the molecular grafting approach utilizing animal-derived macrocyclic, disulfide-rich antimicrobial peptide scaffolds by demonstrating its suitability to obtain ligands with signaling and receptor subtype selectivity, albeit unforeseen.

Pr4LP1 and Ar3LP1 displayed a trend for signaling selectivity by increased efficacy in β-arrestin-2 recruitment assays over Gα_s_ coupling at MC4R, as compared to α-MSH. However, this effect was not significant using the Black–Leff operational model for bias quantification. On the other hand, both peptide ligands did not exhibit any activation of MC1R and MC3R using a cAMP accumulation assay suggesting their receptor subtype selectivity for MC4R. This last point is particularly interesting as setmelanotide has some activity at MC1R that has been reported to drive skin pigmentation.^30^

Without detailed structural studies of ligands in complex with the receptor, it will be difficult to draw any conclusions regarding the structure–activity relationships. However, it is interesting to note that both lead peptides, Pr4LP1 and Ar3LP1, that were selected for detailed pharmacological analysis contained the PHfHW pharmacophore inserted into the antimicrobial peptide scaffolds. Therefore, one might speculate that this motif sequence yields favorable pharmacological properties. On the other hand, the scaffold structure and position of insertion will also have an influence on the efficacy of the grafted peptides. For instance, Eliasen et al., when utilizing the plant-derived cyclotide scaffold, obtained a potent and selective MC4R ligand with the HFRW pharmacophore.^16^

MC4R is of particular interest as a therapeutic target for obesity and metabolic disorders.^31^ MC4R is expressed abundantly in the central and peripheral nervous system and is a G_αs_ protein-coupled receptor that responds to endogenous α- and β-melanocyte-stimulating hormones and can also signal via β-arrestin-dependent pathways in response to agonist stimulation. The hypothalamic expressed MC4R is a critical regulator of energy homeostasis and food intake.^31^ Mutations of the *mc4r* gene have been associated with early onset obesity, hyperphagia, and hyperinsulinemia and have been linked to forms of monogenic and common obesity.^4^ A genome wide association study that involved half a million people from UK Biobank has revealed 61 MC4R variants. Most of these variants caused loss of MC4R function that has been associated with increased risk for obesity and metabolic syndrome.^5^ By contrast, a small subset of gain-of-function MC4R mutations demonstrated protection against obesity, and individuals with these variants demonstrated significantly lower body-mass-index, lower risk for type 2 diabetes and coronary artery disease.^5^ Intriguingly, these protective effects against obesity in people carrying the gain-of-function MC4R variants were driven by signaling bias toward β-arrestin-dependent signaling and mitogen-activated protein kinase pathway activation rather than the canonical G_s_-mediated cAMP pathway. These interesting findings emphasize that development of β-arrestin-biased MC4R ligands may represent an effective strategy for treating weight loss and obesity-related cardiometabolic diseases.^4^ Accordingly, MC4R ligands developed in this study could provide a new platform for the development of functionally selective and safer β-arrestin-biased MC4R agonists for weight loss and metabolic diseases in the future and may cover the substantial and unmet requirements for therapeutics for obesity-related metabolic disorders. Development of biased ligands that may direct signaling toward G protein or arrestin-dependent pathways may allow targeted drug design with desirable pharmacological outcomes, avoiding unwanted off target side effects. In addition, biased ligands can be used as chemical probes to enhance our understanding of MC4R signal transduction to help elucidate the links between signaling and physiological outcomes.

Applying the molecular grafting approach, we have recently demonstrated that the plant-derived SFTI-1 cyclic peptide is a valuable template to develop selective G protein-biased agonists targeting κ-opioid receptor for the treatment of chronic visceral pain without inducing centrally mediated κ-opioid receptor-related adverse effects.^32^ Thus, these studies provide further evidence that grafting bioactive epitopes onto nature-derived cyclic peptide scaffolds is an appealing strategy to fine-tune pharmacology and design receptor subtype and pathway selective ligands that may enable the development of medications with fewer side effects in the future.^5^

In conclusion, our study exemplifies the potential of animal-derived macrocyclic, disulfide-rich antimicrobial peptide scaffolds to design MC4R agonists with improved pathway specificity and receptor subtype selectivity over other melanocortin receptors. The developed MC4R agonists could be valuable probes to delineate MC4R pharmacology, as well as templates to design safer and more effective MC4R-based antiobesity therapeutics in the future. At a more general level, our approach adds toward the GPCR community’s efforts to develop selective ligands for future drug development.^33^

## Experimental Procedures

### Materials

NDP-α-MSH was purchased from Bachem, and α- MSH was obtained from Genscript; the cAMP G_s_ kit was obtained from Cisbio; lipofectamine 3000 was obtained from Fisher Scientific; and furimazine was obtained from Promega or Nanolight Technology, respectively.

### Peptide Synthesis

Peptides were assembled using automated Fmoc solid phase peptide synthesis and rink amide resin on a 0.125 mmol scale. Acetaminomethyl (Acm) and trityl (Trt) were used for orthogonal cysteine (Cys) protection: Acm for Cys3 and Cys16/17, Trt for Cys7 and Cys12/13 for tachyplesin-1; Acm for Cys6 and Cys15/16, Trt for Cys8, and Cys13/14 for protegrin-4; Acm for Cys3 and Cys20/17, Trt for Cys7 and Cys16/13 for arenicin-3; and Acm for Cys2 and Cys15/16, Trt for Cys6, and Cys11/12 for gomesin. Amino acid couplings were performed with four-fold excess of amino acid, four-fold excess of O-(1*H*-6-chlorobenzotriazole-1-yl)-1,1,3,3-tetramethyluronium hexafluorophosphate (HCTU), and eight-fold excess of *N*,*N*-diisopropylethylamine (DIPEA) in dimethylformamide (DMF) for 15 min and repeated twice for each residue. Fmoc was deprotected using 30% piperidine in DMF for 10 min, and the reaction was repeated twice. Peptides were cleaved from the resin with trifluoroacetic acid (TFA)/triisopropylsilane/H_2_O (95:2.5:2.5), precipitated with cold diethyl ether, dissolved in 50% solvent B (90% acetonitrile, 10% water, and 0.05% TFA), and freeze-dried. The four cysteines were then selectively oxidized in a two-pot reaction. First, the nonprotected cysteines were oxidized in 0.1 M ammonium bicarbonate (pH 8–8.5) at a concentration of 0.25 mg/mL and stirred at room temperature for 24 h. Second, the Acm-protected cysteines were oxidized by dissolving the peptides in an iodine solution (0.5 M in acetic acid) at a concentration of 1 mg/mL and stirred for 30 min to 7 h depending on the peptide. Ascorbic acid (10 mg/mL) was added to quench the oxidation, and the solution was stirred until no color was visible. Following cleavage, formation of the first and second disulfide bridge peptides was purified by RP-HPLC on preparative Phenomenex Jupiter C18 columns (5 μm, 300 Å, 250 × 21.2 mm, or 250 × 10 mm) applying a linear gradient from 5 to 65% solvent B (90% ACN, 10% H_2_O, and 0.05% TFA) and flow rates of 8 mL/min. Automatically collected fractions were analyzed by electrospray ionization mass spectrometry and analytical RP-HPLC on a Phenomenex Jupiter C_18_ column (5 μm, 300 Å, 150 × 2 mm) using a linear gradient from 5 to 65% solvent B (Figures S1–S4, Table S1). All peptides displayed a purity of over 95%.

### Cell Culture, Transfection, and Cloning

HEK293T cells were grown at 37 °C (5% CO_2_ atmosphere) and cultured in Dulbecco’s modified Eagle’s medium (DMEM) supplemented with 10% fetal bovine serum and 50–100 U/mL penicillin and 100 U/mL streptomycin. Transient cell transfection was performed with the lipofectamine 3000 transfection reagent as per the manufacturer’s protocol using 2–2.5 μg of total cDNA with the reagent.

### cAMP Second Messenger Quantification Assay

Assays were performed on cells transiently expressing mouse MC4R-EGFP. *Bam*HI and SacI restriction sites were used to clone mouse MC4R N-terminally into a pEGFP-N1 vector. Peptide ligands were assayed for cAMP inhibition in a 384-well format and triplicate according to the Cisbio protocol. Briefly, 5 μL containing 10,000 cells per well was incubated with 5 μL of peptide solutions prepared 2× in 1× stimulation buffer. Following an incubation of peptide ligands at 37 °C for 30 min, d2-labeled cAMP and cryptate-labeled antibody (5 μL of each) were added to the mixture and further incubated for at least 1 h at room temperature. Subsequently, cAMP levels were quantified on a Flexstation 3 (Molecular Devices, San Jose, USA) using homogeneous time-resolved fluorescence resonance energy transfer and the ratio 665/620 nm. Samples were measured in technical triplicates.

### BRET Assay

BRET assay was performed as previously described.^32^ Briefly, β-arrestin-2-nano luciferase and mouse MC4R-EGFP were cotransfected in a 1:10 ratio. 16 h post-transfection, 50,000 cells in 100 μL of phenol red-free DMEM with 10% FBS per well were transferred into white clear bottom 96-well plates and incubated overnight. On the day of the assay, cells were incubated for 1 h at 37 °C in phenol red- and serum-free DMEM. Furimazine (diluted 1:50) and peptide ligands (4×) were prepared in Hank’s balanced salt solution (HBSS), respectively. Prior to establishing the baseline and measuring kinetic BRET, furimazine was incubated for 5 min at 37 °C. The ability of peptide ligands to induce β-arrestin-2 recruitment was assessed by measuring light emissions for EGFP (510 nm) and nanoluciferase (460 nm) over 35 min using Flexstation 3 (Molecular Devices, San Jose, USA). The BRET ratio was calculated as emission EGFP (510 nm)/emission nanoluciferase (460 nm). The ligand-induced BRET was calculated by subtracting the BRET ratio of the ligand from the BRET ratio of the vehicle control (HBSS).

### Luciferase Complementation Assays

The plasmid constructs used to show interactions between human MC4R and G_αs_ protein and/or β-arrestin-2 were designed with fused SmBit and LgBit proteins, that upon complementation form the fully functional nanoluciferase enzyme that can produce detectable bioluminescence upon addition of the substrate furimazine. For the Gαs coupling, we used a LgBit fused at the C-terminus of MC4R (MC4R-LgBit) and cotransfected with the G_αs_-SmBit construct. For the β-arrestin-2 recruitment assay, we used a C-terminally fused MC4R-SmBit and the β-arrestin-2-LgBit constructs. The cotransfection ratio was 1:10 (G_αs_/β-arrestin-2/receptor). Cells were harvested, washed, and resuspended in fresh medium 24 h post-transfection. Approximately 5 × 10^4^ cells/well were distributed in precoated 96-well white plates and allowed to adhere overnight. On an experimental day, cells were washed with HBSS and maintained in HBSS (80 μL/well). The furimazine substrate for the luciferase (50×) stock was diluted at 1× with HBSS, and 10 μL/well was added to all assay wells and incubated for 12 min before addition of ligands. Ligand dilutions were prepared at 10× final concentration in HBSS, and 10 μL/well was added to the assay wells. Plates were read 5 min following ligand addition using a ClarioStar equipped with a nanoluc-compatible optic module (BMG Labtech). The data were initially normalized, in excel, to vehicle control (HBSS) and then further normalized to α-MSH maximum response to define the minimum and maximum response to the endogenous ligand. EC_50_/*E*_max_ values were calculated via a nonlinear regression analysis.

### pGlo cAMP Accumulation Assay

Cells transiently expressing human MC1R, MC3R, or MC4R were incubated for 24 h. Afterward all contents of each well were carefully removed, and cells were then equilibrated in cAMP buffer [1× HBSS, 24 mM HEPES, 0.1% (w/v) BSA, 3.96 mM NaHCO_3_, 1 mM MgSO_4_, 1.3 mM CaCl_2_ dihydrate], supplemented with firefly d-luciferin free acid (0.45 mg/mL) (NanoLight Technology). 90 μL of cAMP buffer was added to all wells, and the 96-well plate was then incubated (28 °C, 1 h). Bioluminescence was measured using the CLARIOstar Plus Plate Reader (BMG LabTech). Prior to injecting the treatment ligands, approximately 5–10 basal readings were performed until stabilization was reached. Peptide ligands (10 μL) were added to the well. Bioluminescence was measured for a total of 30 cycles (1 min per cycle, 1 s integration time, without filter, a fixed gain of 3000, and autofocus).

### Data Analysis

Data analysis was performed with GraphPad Prism (GraphPad Software, San Diego). The potency (EC_50_) and maximum efficacy (*E*_max_) values of peptide ligands were derived from functional data fitted to three- or four-parameter nonlinear regression curves constrained to a bottom of zero. Except those otherwise mentioned, graphs were normalized to 100%, i.e., the highest concentration of NDP-α-MSH or α-MSH.

## Supporting Information Available

HPLC quality control of MC4R peptide analogues; β-arrestin-2 recruitment data of MC4R peptide analogues; and mass spectrometry data of MC4R peptide analogues (PDF)
Raw, background subtracted measurements of the BRET kinetic experiments (XLSX)
