# Supplementary material for: Development of Melanocortin 4 Receptor Agonists by Exploiting Animal-Derived Macrocyclic, Disulfide-Rich Peptide Scaffolds
Source: ACS Pharmacol Transl Sci. 2023 Sep 26;6(10):1373–81. doi: 10.1021/acsptsci.3c00090 (PMC10580383; doi:10.1021/acsptsci.3c00090)
Supplement: Supplementary file 1 — pt3c00090_si_001.pdf [file pt3c00090_si_001.pdf]

Supplementary Information

Development of melanocortin 4 receptor agonists by exploiting animal-derived macrocyclic, disulfide-rich peptide scaffolds

Edin Muratspahić[1,2,#], Despoina Aslanoglou[3,#], Andrew M. White[2], Claudia Draxler[1], Xaver Kozisek[1], Zara Farooq[3], David J. Craik[2], Peter J. McCormick[3], Thomas Durek[2], Christian W. Gruber[1]*

[1]Center for Physiology and Pharmacology, Institute of Pharmacology, Medical University of Vienna, 1090 Vienna, Austria

[2]Institute for Molecular Bioscience, Australian Research Council Centre of Excellence for Innovations in Peptide and Protein Science, The University of Queensland, Brisbane, Queensland 4072, Australia

[3]Department of Endocrinology, Queen Mary University of London, London E1 4NS, United Kingdom

[#]These authors contributed equally.

*For correspondence: Christian W. Gruber, christian.w.gruber@meduniwien.ac.at

This pdf contains:

Figures S1-S5

Table S1

Supplied as separate file:

Data S1

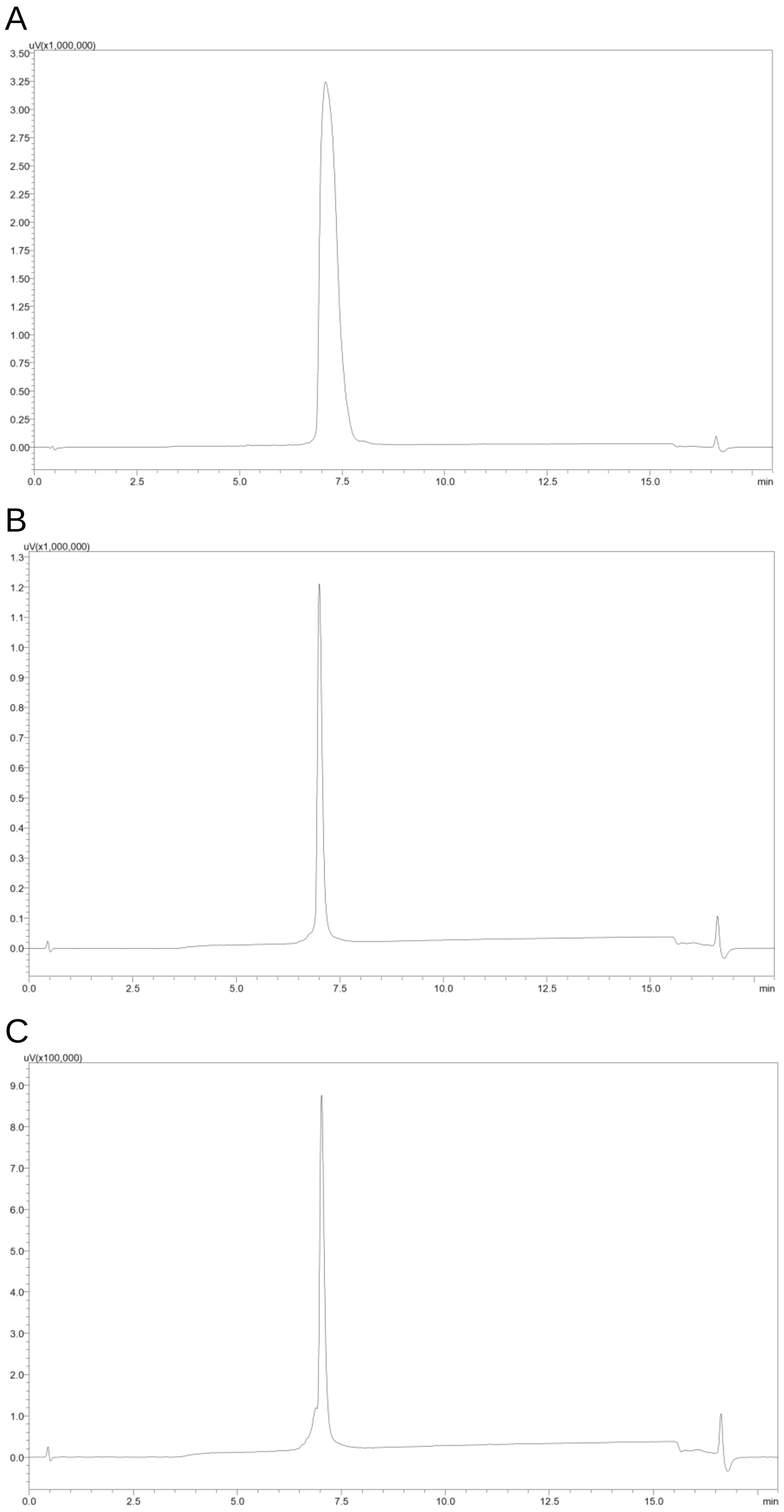

Figure S1. HPLC of Ta1LP1 (top, A), Ta1LP2 (middle, B), Ta1LP3 (bottom, C). Linear gradient with 5-65% solvent B over 15 min and a flow rate of 0.6 ml/min on a C_{18} column (5 µm, 300 Å, 150 × 2 mm) was applied.

A

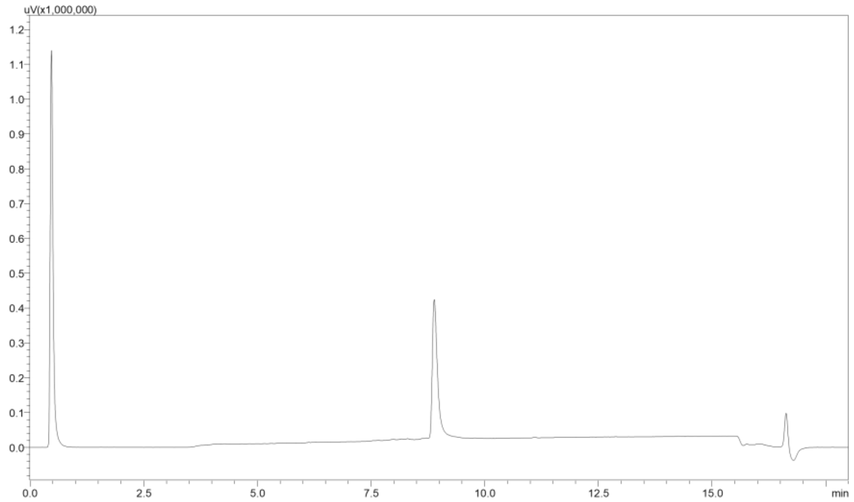

B

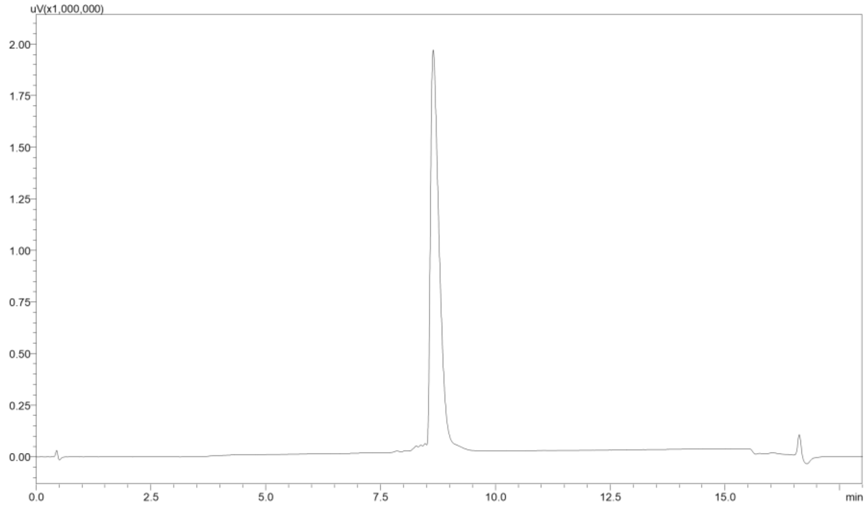

C

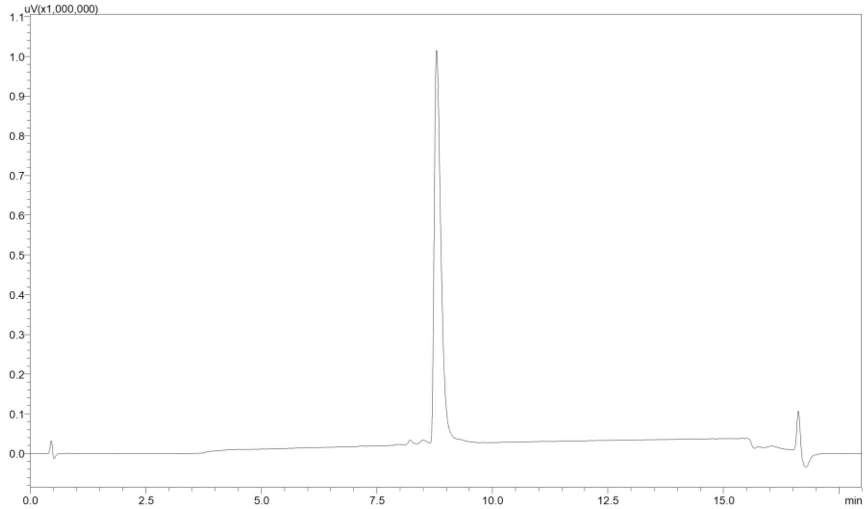

Figure S2. **HPLC of Pr4LP1 (top, A), Pr4LP2 (middle, B), Pr4LP3 (bottom, C).** Linear gradient with 5-65% solvent B over 15 min and a flow rate of 0.6 ml/min on a C_{18} column (5 µm, 300 Å, 150 × 2 mm) was applied.

A

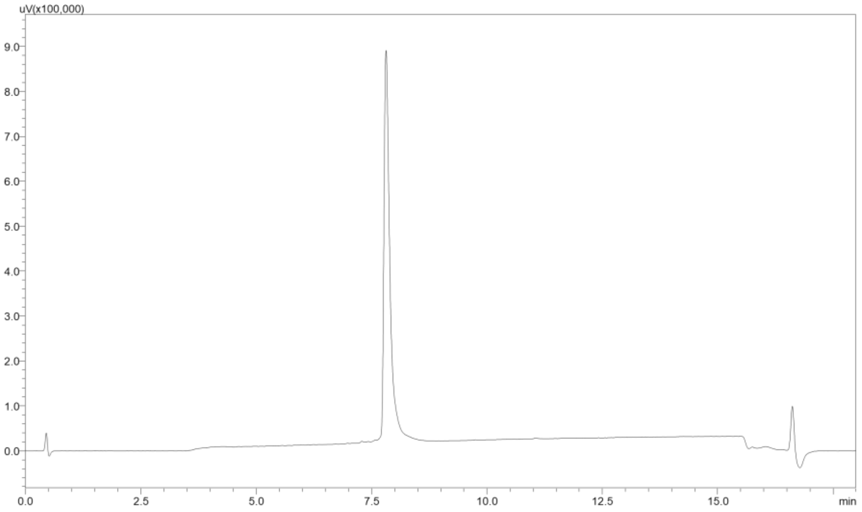
uV(x100,000)
9.0
8.0
7.0
6.0
5.0
4.0
3.0
2.0
1.0
0.0
0.0
2.5
5.0
7.5
10.0
12.5
15.0
min

B

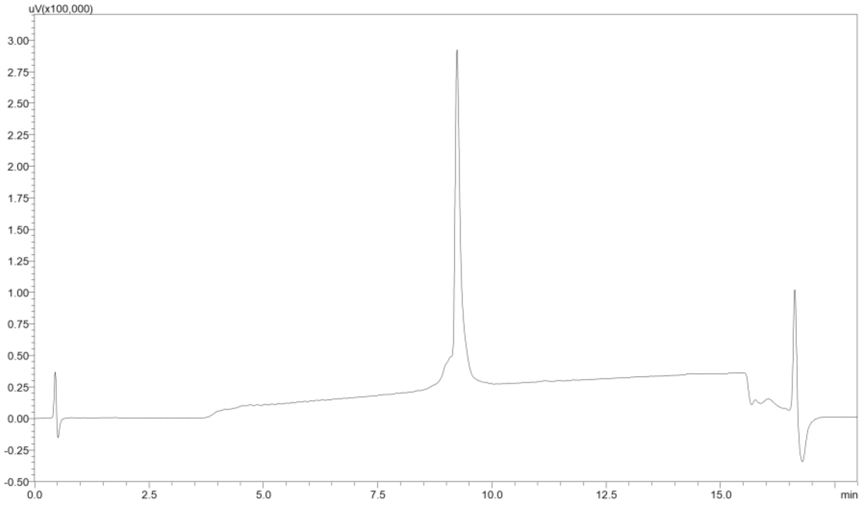
uV(x100,000)
3.00
2.75
2.50
2.25
2.00
1.75
1.50
1.25
1.00
0.75
0.50
0.25
0.00
-0.25
-0.50
0.0
2.5
5.0
7.5
10.0
12.5
15.0
min

C

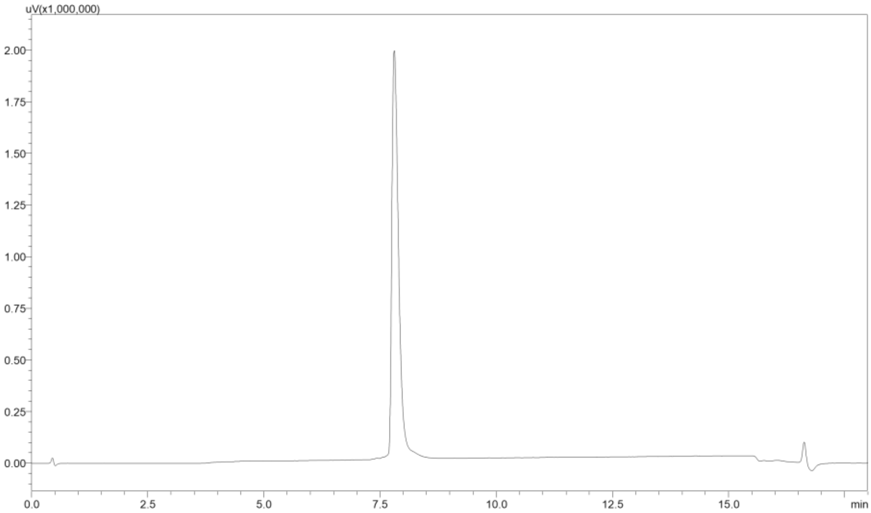
uV(x1,000,000)
2.00
1.75
1.50
1.25
1.00
0.75
0.50
0.25
0.00
0.0
2.5
5.0
7.5
10.0
12.5
15.0
min

D

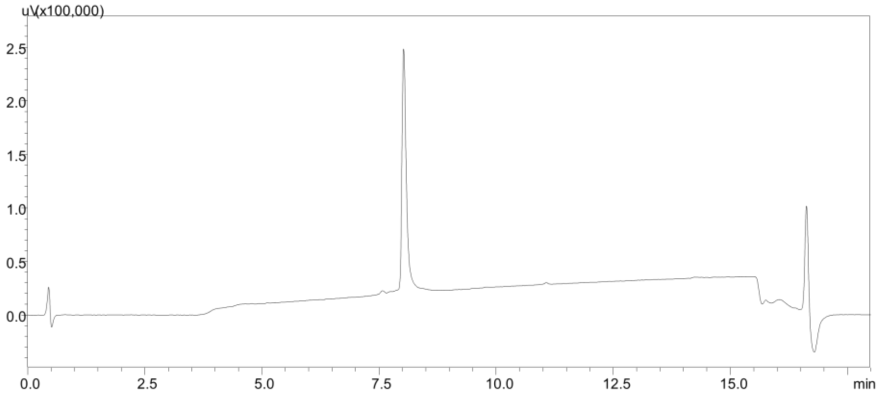

E

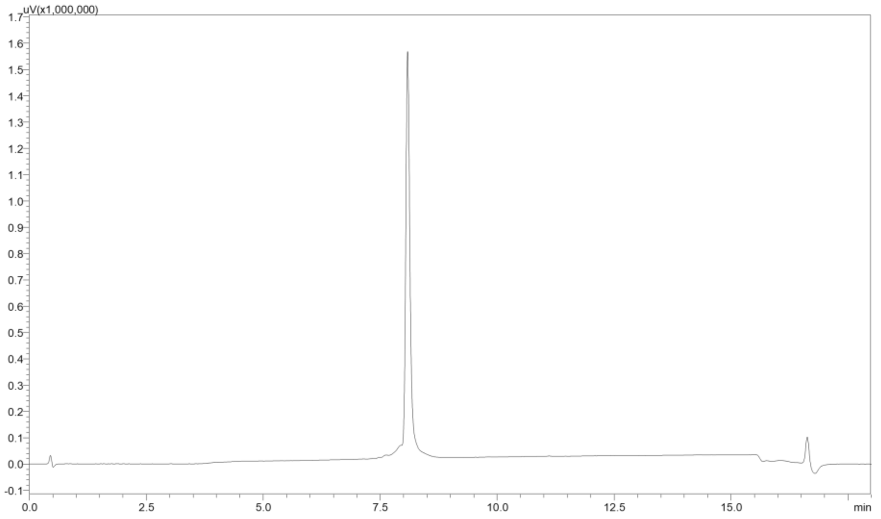

Figure S3. HPLC of Ar3LP1 (top, A), Ar3LP2 (middle, B), Ar3LP3 (bottom, C), Ar3LP4 (top, D) and Ar3LP5 (bottom, E). Linear gradient with 5-65% solvent B over 15 min and a flow rate of 0.6 ml/min on a C_{18} column (5 µm, 300 Å, 150 × 2 mm) was applied.

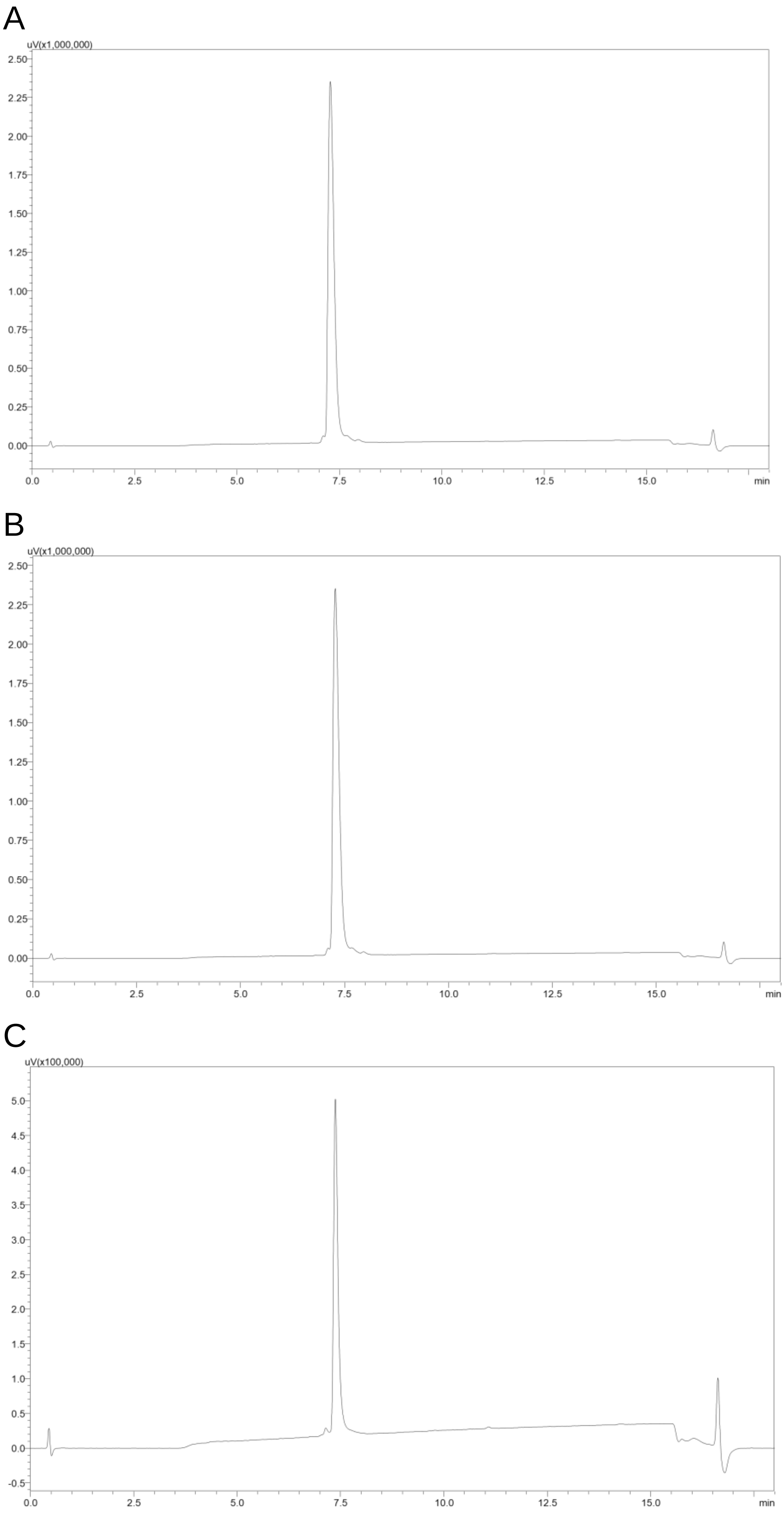

Figure S4. HPLC of GoLP1 (top, A), GoLP2 (middle, B), GoLP3 (bottom, C). Linear gradient with 5-65% solvent B over 15 min and a flow rate of 0.6 ml/min on a C_{18} column (5 μm, 300 Å, 150 × 2 mm) was applied.

Table S1. Mass spectrometry data of synthesized MC4R peptide analogs.

Peptide	Calculated mass (m/z)	Observed mass (m/z)
Ta1LP1	826.7	827.2
Ta1LP2	794.4	794.8
Ta1LP3	800.7	801.1
Pr4LP1	764.7	765.1
Pr4LP2	732.3	732.8
Pr4LP3	738.7	739.1
Ar3LP1	791.3	791.8
Ar3LP2	911.7	912.3
Ar3LP3	909.4	909.9
Ar3LP4	931.4	931.9
Ar3LP5	937.8	938.3
GoLP1	800.4	800.8
GoLP2	768.0	768.4
GoLP3	774.4	774.8

Masses of peptides ($[M+2H]^{2+}$) measured by electrospray ionization-mass spectrometry (ESI-MS) are shown.

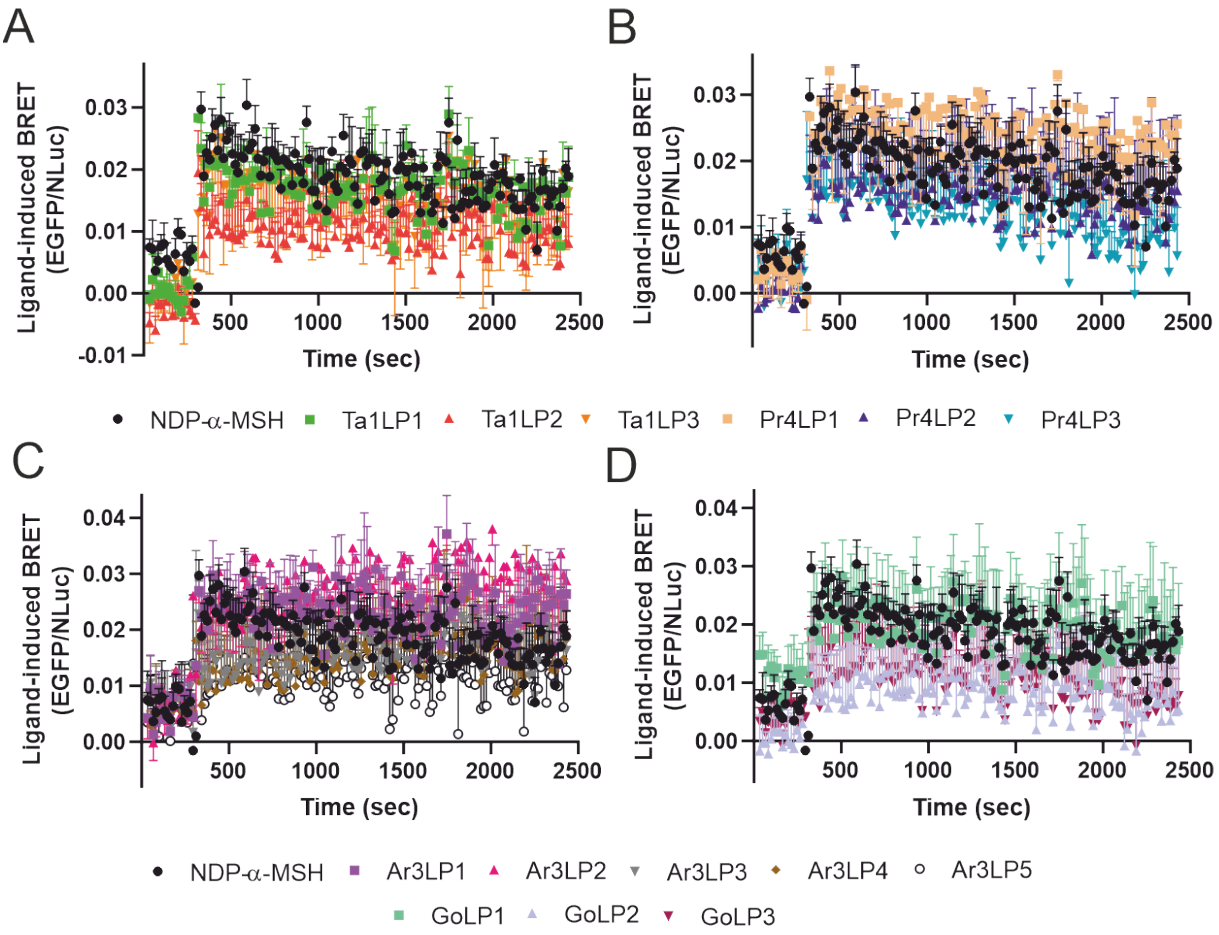

Figure S5. β-arrestin-2 recruitment data of MC4R peptide analogs. Bioluminescence resonance energy transfer (BRET) assay was used to measure the transient interaction of MC4R-EGFP with β-arrestin-2-nanoluciferase (NLuc) in HEK293 cells over time following cell treatment with 10 µM of **A**, tachyplesin-1-like peptides (Ta1LPs), **B**, protegrin-4-like peptides (Pr4LPs), **C**, arenicin-3-like peptides (Ar3LPs) and **D**, gomesin-like peptides (GoLPs). Data are shown as mean ± SEM from three independent experiments. Raw mean data (HBSS buffer subtracted) of these graphs have been supplied as Excel file (**Data S1**).
